# The Impact of an Acute Care Surgical Service on the Quality and Efficiency of Care Outcome Indicators for Patients with General Surgical Emergencies

**DOI:** 10.7759/cureus.5036

**Published:** 2019-06-29

**Authors:** Krista Hardy, Jennifer Metcalfe, Kathleen Clouston, Ashley Vergis

**Affiliations:** 1 Surgery, St. Boniface Hospital, University of Manitoba, Winnipeg, CAN

**Keywords:** acute care surgery service, retrospective chart review, patient and health system outcomes, acute appendicitis, acute cholecystectomy

## Abstract

Background

Acute care surgery (ACS) models address high volumes of emergency general surgery and emergency room (ER) overcrowding. The impact of ACS service model implementation on the quality and efficiency of care (EOC) outcomes in acute appendicitis (AA) and acute cholecystitis (AC) cohorts was evaluated.

Methods

A retrospective chart review (N=1,229) of adult AA and AC patients admitted prior to (pre-ACS; n=507; three hospitals; 2007) and after regionalization (R-ACS; n=722; one hospital; 2011).

Results

R-ACS time to ER physician assessment was significantly longer for AA (3.4 ± 2.3 versus 2.4 ± 2.6 hr; p ≤ 0.001). Surgical response times (1.3 ± 1.2 vs 2.6 ± 4.3 hr for AA; 1.8 ± 1.5 vs 4.1 ± 5.0 hr for AC; p ≤ 0.0001) and acquisition of imaging (4.1 ± 4.1 vs 6.9 ± 9.9 hr for AA, p ≤ 0.0001; 7.8 ± 1.9 vs 13.2 ± 18.5 hr for AC, p ≤ 0.008) occurred significantly faster with R-ACS. R-ACS resulted in a significant increase in night-time appendectomies (21.7% vs 11.1%; p ≤ 0.002), perforated appendices (29.1 % vs 18.9 %; p ≤ 0.006), 30-day readmissions (4.56% vs 0.82%; p ≤ 0.01), and lower rate of intraoperative complications for AC patients (2.78% vs 7.69%; p ≤ 0.02).

Conclusions

Despite the increased volume of patients seen with the implementation of R-ACS, surgical assessments and diagnostic imaging were significantly more prompt. EOC measures were maintained. Worse AA outcomes highlight areas for improvement in delivering R-ACS.

## Introduction

General surgeons face the challenge of managing high volumes of emergency general surgery (EGS) patients with advanced age and increasingly complex conditions [1]. Often, urgent intervention to prevent rapid patient deterioration is necessary [2] and compounded by limits in emergency department (ED) access due to overcrowding. Such delays may be associated with higher rates of major complications and death [3]. ACS was developed as an extension of trauma surgery services, including emergency surgery while maintaining operative skills through increased operative volume. It also benefited patients by improving the timeliness of care [4]. In Canada, the impetus to create acute care surgery services (ACS) also arose from the high volume of EGS cases, increasing surgeon sub-specialization, facilitation of the separation of elective practice from emergency surgery cases, and the regionalization of subspecialty surgical care [5].

An organized ACS team is proposed to facilitate the prompt, comprehensive, evidence-based care of EGS patients, leading to improved efficiency of care (EOC) measures, reduced complications, and decreased length of stay and cost [6-16]. A dedicated service with protected daytime operating and a surgeon relieved of elective duties should facilitate more daytime operating [13-14,16] and potentially minimize human error related to surgeon fatigue [7].

There are 13 ACS services in Canada with significant variation in organization and function [1,5]. The lack of standardization for ACS outcome measures and reporting makes comparisons among ACS services challenging [17-18]. Acute appendicitis (AA) and acute cholecystitis (AC) are the most common EGS diagnoses requiring admission [7], with standard, measurable points of care along the patient trajectory.

The lack of agreement and utilization of standard metrics to report EOC and patient outcomes within ACS service delivery, in addition to the high degree of variability in the definitions of each variable, make reporting and comparisons challenging. Standardization and consensus is required among ACS surgeons (and the health care teams/stakeholders involved in delivering care) to create an organized system of data capture and analysis to establish national standards for ACS service implementation and delivery, including support for evidence-based practice, quality improvement initiatives, as well as research, training/education, and innovation. Metcalfe et al. [17] reported that approximately 27% (6/22) of studies of ACS implementation provided surgical response time and included five different definitions for this metric. Time to operating room was reported in 91% (20/22) of studies, with between five to seven distinct definitions while LOS was reported in 86% of studies with three separate definitions.

Three Winnipeg hospitals’ EGS services were consolidated into a regionalized ACS service (R-ACS) [6], resulting in a 221% increase in patient volume. Approximately 56% of ACS service in-patient admissions are transferred from another institution; 39 provincial hospitals (five within and 34 outside Winnipeg), as well as 19 other institutions (nursing stations, personal care homes, health centers, home care/physician’s offices). Interhospital transfer has been shown to impact the surgical quality and patient outcome metrics, as well as the utilization of hospital and health system delivery resources [19-20].

The average annual number of cases admitted to the ACS service was 4,024; depending on the hospital, between 32% to 53% are medically managed while 47% to 68% are surgically managed. Per capita, the Winnipeg ACS service receives approximately 566 ACS admissions per 100,000 people. Research evaluating whether R-ACS in Winnipeg leads to improved efficiency and patient outcomes is crucial in facilitating evidence-based care, creating benchmark standards, and highlighting areas for system improvement. The study objective was to determine the impact of implementing a regionalized ACS on EOC and patient outcomes for common general surgical emergencies (acute appendicitis and acute cholecystitis).

## Materials and methods

**Figure 1:**
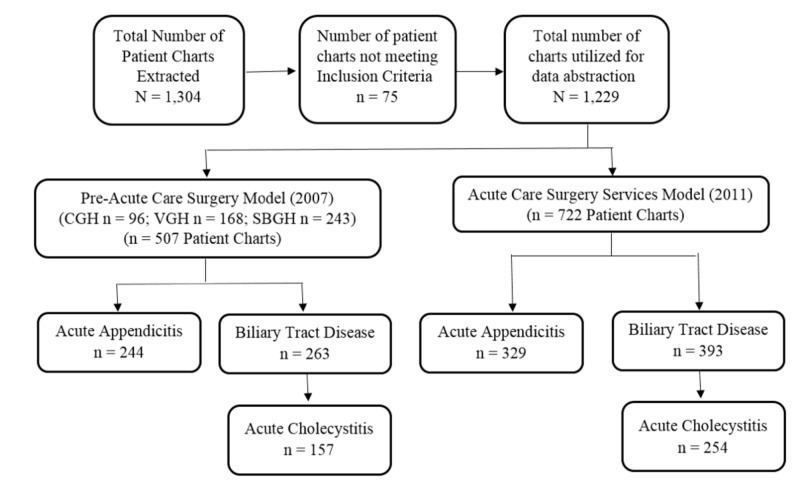
Flow Diagram of Patient Chart Extraction Process, Number of Charts for Data Abstraction, and Study Results for the Pre-Acute Care (Pre-ACS; 2007) and Acute Care Surgery (ACS; 2011) Service Models *STROBE (The Strengthening the Reporting of Observational Studies in Epidemiology) Guidelines [8]

A retrospective chart review of AA and AC patients was performed to compare EOC and patient outcome measures between the Pre-ACS and R-ACS cohorts (University of Manitoba Health Research Ethics Board number H2012:166; HS15311; individual institutional ethics approvals obtained). A flow diagram of the patient chart extraction process is provided in Figure 1. AA (n=244 and n=329 for the Pre-ACS and R-ACS groups, respectively) and AC (n=157 and n=254 for the Pre-ACS and R-ACS groups, respectively) diagnoses were chosen, as they are the most common reasons for EGS admission [7] with standard, measurable points of care. Adult (>17 years) patients diagnosed with AA or AC during the January to December (2007) and January to December (2011) study periods were included. Charts were identified by diagnostic and procedural ICD-10 codes and included regardless of whether or not they underwent an operation during admission. Exclusion criteria included patients who were admitted to a service other than general surgery or ACS, admission with a diagnosis not meeting the case definition, and patients undergoing appendectomy or cholecystectomy as an elective procedure or secondary to another operation. Biliary cases with a diagnosis other than acute cholecystitis were excluded (including gallstones, choledocholithiasis, and biliary colic) due to the significant variability in course of care and surgical decision-making.

Three hospitals in Winnipeg (Canada) contributed to this investigation: St. Boniface General Hospital (tertiary academic), Concordia General Hospital (urban community), and Victoria General Hospital (urban community). The Pre-ACS implementation period from January 1 to December 31, 2007, represented the traditional “on-call” model. This cohort included patients admitted emergently to the general surgery services of each hospital. The ACS service was implemented in 2008 and the regionalized or R-ACS study period was January 1 to December 31, 2011. This year was selected for R-ACS to represent a stable mature system that had been functioning for several years. In the regionalized model, patients from the Concordia and Victoria General Hospitals were routinely transferred to St. Boniface for evaluation. During this period, there was a dedicated weekday operating room time designated for emergency general surgery patients (1000 hrs to 1530 hrs). Each of the three Pre-ACS hospitals had their own ‘on-call’ roster servicing their emergency department that acted independently and was staffed by a single surgeon (urban community sites) or a single surgeon/resident team (SBGH; tertiary). The surgeon/resident team took EGS calls, had regular elective duties, and completed their EGS responsibilities on an ad hoc basis.

**Figure 2:**
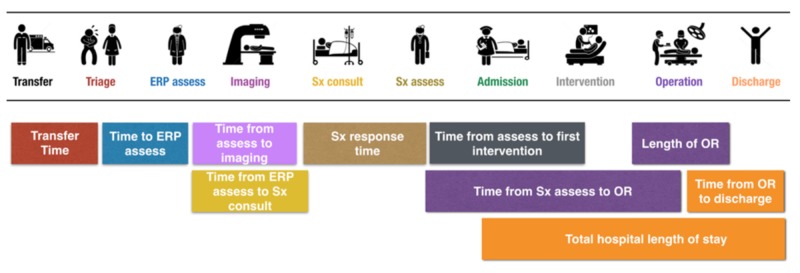
Efficiency of Care Time Variables *ERP, emergency room physician; Sx, Surgical assessment; OR, operating room.

Patient baseline demographics were recorded, including age at admission (years), gender, body mass index (BMI), previous laparotomy, smoking status, medications (anticoagulants and steroids), and the following five comorbidities: (1) Cardiac (ischemic heart disease, arrhythmia, congestive heart failure), (2) Respiratory (chronic obstructive pulmonary disease (COPD), asthma), (3) Bleeding diathesis (bleeding disorder, cirrhosis), (4) Endocrine (diabetes mellitus), (5) Hematology (deep vein thrombosis, pulmonary embolism). The existence of these comorbidities was important to document, as they may impact the peri-operative variables under investigation. Data for the Pre-ACS and R-ACS periods were collected for multiple EOC time points along the patient trajectory (admission to discharge), allowing a retrospective temporal trend analysis of the data (similar to interrupted time series analysis). Both date and time were recorded for the general surgery/ACS consultation (origin hospital), triage/assessment room, emergency room physician assessment, surgical consultation, surgical assessment, hospital admission, and hospital discharge EOC variables. Date, time, and location were recorded for the radiology (ultrasound, computed tomography, and magnetic resonance imaging) and non-operative intervention (endoscopic retrograde cholangiopancreatography (ERCP), percutaneous drain insertion, gastroscopy, and colonoscopy) EOC variables. Start time (time of surgical incision), end time (skin closure), time of day (day (07:00-15:59); evening (16:00-23:59), or night (00:00-06:59)), surgical procedure type/classification (laparoscopic, open or converted) were recorded for the operative intervention EOC variable. The EOC time variables are depicted in Figure 2. Five baseline hospital admission variables were measured, including (1) admission diagnosis, (2) origin hospital (if transferred), (3) Regional Health Authority of the origin hospital, (4) admission hospital and service, and (5) previous admissions information for the same diagnosis.

The patient outcome variables measured included intra-operative, intervention-related, and postoperative complications, time to operating room, total length of stay (LOS), 30-day hospital readmission rate, 30-day emergency room (ER) visits, risk of perforated appendicitis, and pathology for appendectomy and cholecystectomy specimens. Post-operative complications were graded according to the Clavien-Dindo Classification System on a scale of I through V; minor grades I to II, major grades III to V [9].

Study-specific reference documents, specifying the chart location for each outcome variable being extracted, facilitated reproducible and uniform data extraction (Supplemental Files 1, 2, and 3 in the Appendix). If the variable was not found in the pre-specified location, it was recorded as “not specified.” Uniform and consistent data extraction methods were facilitated through joint data extracted by two research team members (HG and JM); 50 charts at both Concordia and Victoria General Hospitals. Where differences in assessment existed, discussion and resolution were attained and, if required, arbitrated by KH. SAS procedures were utilized to handle missing data. For data missing completely at random, listwise deletion was used (the number of missing values was low). Data missing at random were dealt with through imputation.

A sample size/power calculation was not performed for this research study, as all of the charts for each cohort (Pre-ACS and R-ACS) for both the acute appendicitis (AA) and acute cholecystitis (AC) groups were retrieved and reviewed.

Data were analyzed with SAS® statistical software (SAS Institute, North Carolina, US). The AA and AC groups within the Pre-ACS and R-ACS cohorts were analyzed separately. Continuous data were analyzed with the two-tailed student’s t-test. Chi-square tests were used to test categorical variables; Fisher’s exact test was used if sample numbers were small (<5). A logistic regression analysis was performed for postoperative complications, readmissions, risk of perforated appendicitis, time to OR, and total length of stay (LOS). Logistic regression model covariates were chosen by a clinical decision and the backward selection method. Age and gender were deemed significantly relevant and included in all logistic models. The backward selection method started with all covariates included in the analysis plan. Variables were deleted from the model based on p-value; p-values > 0.02 were deleted. Cook’s D statistics were plotted for each regression model to determine the influence of each observation. Tolerance values to test for multicollinearity among the covariates was performed and absence confirmed among the variables tested; all tolerance values were greater than 0.1 and had Variance Inflation Factor (VIF) less than 10.

## Results

Data were extracted from a total of 1,229 charts and represents two cohorts: the Pre-ACS (n=507) and R-ACS (n=722) cohorts. The Pre-ACS cohort includes patients from three hospitals (n=243 SBGH, n=168 VGH, n=96 CGH). The AA and AC procedures were utilized to compare the Pre-ACS and ACS surgical outcomes. Table 1 outlines the baseline demographic variables for both AA and AC groups for the Pre-ACS versus R-ACS cohorts. No significant differences were found in the baseline demographic variables between the Pre-ACS and R-ACS cohorts for AC. All baseline demographic variable outcomes were similar for AA patients in both the Pre- and R-ACS cohorts, except mean age at admission, which was significantly higher in the R-ACS group as compared to the Pre-ACS group (39.26 ± 16.63 vs 36.35 ± 15.05; p<0.03) and a significantly larger percentage of patients in the R-ACS cohort had a previous midline laparotomy compared to the Pre-ACS cohort (5.18% vs 1.23%; p<0.01). The presence of the five comorbidities was similar between the Pre- and R-ACS cohorts for both the AA and AC groups (Table 2).

**Table 1:** Baseline Demographics for Acute Appendicitis (AA) and Acute Cholecystitis (AC) in the Pre-ACS (2007) versus Regionalized ACS (2011) Cohorts Note: ¹indicates sample size of 130, ²indicates sample size of 309,^ 3^indicates sample size of 89, ^4^indicates sample size of 239; *indicates a Fisher’s Exact Test was used; R-ACS is Regionalized Acute Care Surgery (ACS); Select medications are steroids and anticoagulants.

Variable	Acute Appendicitis			Acute Cholecystitis		
	Pre-ACS (n = 244)	R-ACS (n = 329)	p-value	Pre-ACS (n = 157)	R-ACS (n = 254)	p-value
Mean Age (SD)	36.4 (15.1)	39.3 (16.6)	0.03	54.8 (18.4)	51.3 (18.2)	0.06
Gender (%)						
Male	51.2	52.4	0.7	37.8	41.3	0.5
Female	48.8	47.6		62.2	58.7	
Mean BMI (SD)	27.5 (6.2)¹	26.4 (5.4)²	0.06	29.5 (6.45)^3^	30.5 (6.78)^4^	0.2
Previous Laparotomy (%)						
No	98.8	94.8	0.01*	95.5	91.7	0.1
Yes	1.2	5.2		4.5	8.3	
Select Medications (%)						
No	97.5	98.8	0.3*	91.7	94.8	0.2
Yes	2.5	1.2		8.3	5.2	

**Table 2:** Comorbidities for Acute Appendicitis (AA) and Acute Cholecystitis (AC) in the Pre-ACS (2007) versus Regionalized ACS (2011) Cohorts *indicates a Fisher’s Exact Test was used.

Comorbidity	Acute Appendicitis			Acute Cholecystitis		
	Pre-ACS (2007)	R-ACS (2011)	p-value	Pre-ACS (2007)	R-ACS (2011)	p-value
Cardiac						
Yes	3.3	2.7	0.7	13.6	10.1	0.4
No	96.7	97.3		86.4	89.9	
Respiratory						
Yes	2.9	3.7	0.6	4.6	2.1	0.3
No	97.1	96.4		95.5	97.9	
Bleeding Diathesis						
Yes	0.8	0.0	0.2*	0.00	1.1	0.5
No	99.2	100.0		100.0	98.9	
Hematology						
Yes	1.2	0.3	0.3*	0.9	0.53	1.00*
No	98.8	99.7		99.1	99.5	
Endocrine						
Yes	-	-	-	8.2	11.6	0.3
No	-	-		91.8	88.4	

**Figure 3:**
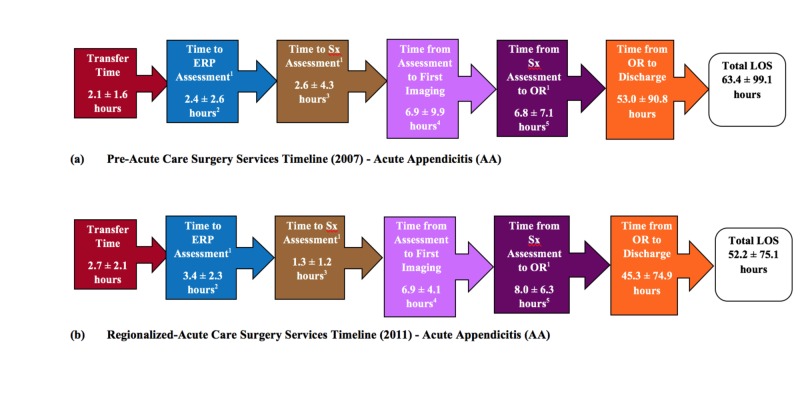
Time Line by Stage Between ER Arrival and Operative Intervention for Pre- and Regionalized ACS Services Cohorts: Acute Appendicitis (AA) ^1^ERP, emergency room physician; Sx, Surgical assessment; OR, operating room; ACS: acute care surgery. ^2^p<0.001; 3 p<0.0001; ^4^p<0.0001; ^5^p<0.03.

**Figure 4:**
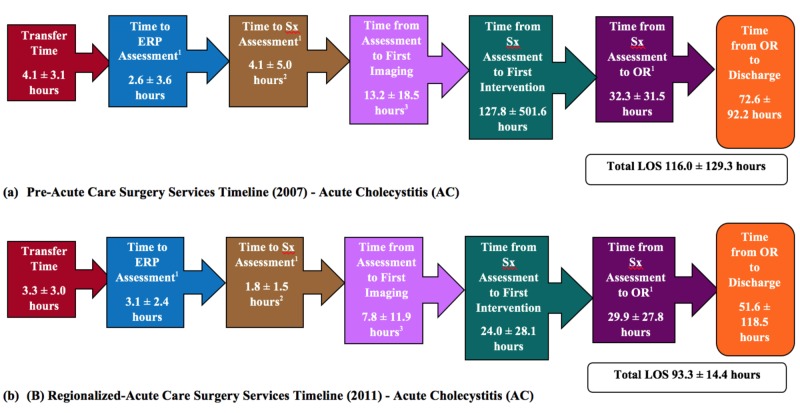
Time Line by Stage Between ER Arrival and Operative Intervention for Pre- and Regionalized ACS Services Cohorts: Acute Cholecystitis (AC) ^1^ERP, emergency room physician; Sx, Surgical assessment; OR, operating room; ACS: acute care surgery. ^2^p<0.0001; 3p<0.01

EOC and patient outcome measures were compared between the Pre-ACS and R-ACS cohorts for both the AA and AC patient groups (Table 3 and Table 4; Figure 3 and Figure 4). For both the AA and AC diagnoses, the R-ACS cohort had a significantly higher percentage of hospital transfers as compared to direct admissions (AA=67.1% vs 14.3%, p<0.0001; AC=59.5% vs 14.2 %, p<0.0001) and laparoscopic procedures (versus open; AA=97.8% vs 80.6%, p<0.0001; AC=100% vs 94.4%, p<0.0001). The mean length of time for AA and AC procedures were significantly longer in the R-ACS service cohort as compared to the Pre-ACS cohort (AA=62.8 vs 55.5 minutes, p<0.001; AC=96.7 vs 82.5 minutes, p<0.0001). Significantly fewer R-ACS cohort AA patients had their surgeries during the evening (16:00 to 23:59) as compared to the Pre-ACS cohort AA patients (50.3% vs 63.2%, p<0.002) while a significantly larger percentage of R-ACS patients, as compared to Pre-ACS patients, had their surgery at night (00:00 to 06:59; 21.7% vs 11.1%, p<0.002). A similar number of AA patients had their surgeries during the day in both the Pre-ACS and ACS cohorts. No differences in surgical time of day existed between the cohorts for AC patients. For non-operative interventions, three percutaneous drain insertions were performed (two of the interventions involving the same patient) in the Pre-ACS AA group. One patient in the R-ACS AA patient group was managed non-operatively (missed appendicitis with phlegmon). For both the Pre- and R-ACS cohorts, ERCP was the most common non-operative intervention performed in the AC patient group and did not differ between the cohorts (22.4 versus 15.3 %; p=0.08). In addition, no significant differences were found in the frequency of operation for acute cholecystitis among the Pre-ACS and R-ACS cohorts (90.3% versus 94.4%, p=0.10).

**Table 3:** Efficiency of Care Outcome Variables for Acute Appendicitis and Acute Cholecystitis in the Pre-ACS (2007) versus Regionalized ACS (2011) Cohorts ^1^Abbreviations: LOS=length of stay; ERP=emergency room physician; Sx=surgeon; assess=assessment; OR=operation; min=minutes; SD=standard deviation; R-ACS=Regionalized Acute Care Surgery ^2^Sample sizes varied between the cohorts for each outcome variable assessed for AA. Transfer time (n=32 and 213 for Pre-ACS and R-ACS, respectively); Total LOS (n=243 and 329 for Pre-ACS and R-ACS, respectively); Time to ERP assess (n=197 and 109 for Pre-ACS and R-ACS, respectively); Time to Sx assess (n=205 and 307 for Pre-ACS and R-ACS, respectively); Time from ERP or Sx assess to first imaging (n=146 and 181 for Pre-ACS and R-ACS, respectively); Time from Sx assess to OR (n=230 and 328 for Pre-ACS and R-ACS, respectively); Time from OR to discharge (n=238 and 328 for Pre-ACS and R-ACS, respectively). ^3^Sample sizes varied between the cohorts for each outcome variable assessed for AC. Transfer time (n=23 and 153 for Pre-ACS and R-ACS, respectively); Total LOS (n=175 and 267 for Pre-ACS and R-ACS, respectively); Time to ERP assess (n=144 and 105 for Pre-ACS and R-ACS, respectively); Time to Sx assess (n=144 and 244 for Pre-ACS and R-ACS, respectively); Time from ERP or Sx assess to first imaging (n=116 and 122 for Pre-ACS and R-ACS, respectively); Time from Sx assess to OR (n=151 and 252 for Pre-ACS and R-ACS, respectively); Time from OR to discharge (n=158 and 252 for Pre-ACS and R-ACS, respectively); Time from Sx assess to first intervention (n=27 and 61 for Pre-ACS and R-ACS, respectively).

Variable^1^	Acute Appendicitis (AA)^2^			Acute Cholecystitis (AC)^3^		
	Pre-ACS Mean Hours (SD)	R-ACS Mean Hours (SD)	p-value	Pre-ACS Mean Hours (SD)	R-ACS Mean Hours (SD)	p-value
Transfer Time	2.1 (1.6)	2.7 (2.1)	0.2	4.1 (3.1)	3.3 (3.0)	0.3
Total LOS	63.4 (99.1)	52.2 (75.1)	0.1	116.0 (129.3)	93.3 (144.4)	0.1
Time to ERP assess	2.4 (2.6)	3.4 (2.3)	0.001	2.6 (3.6)	3.1 (2.4)	0.2
Time to Sx assess	2.6 (4.3)	1.3 (1.2)	<0.0001	4.1 (5.0)	1.8 (1.5)	<0.0001
Time from ERP or Sx assess to first imaging	6.9 (9.9)	4.1 (4.1)	<0.0001	13.2 (18.5)	7.8 (11.9)	0.01
Time from Sx assess to OR	6.8 (7.1)	8.0 (6.3)	0.03	32.3 (31.5)	29.9 (27.8)	0.4
Time from OR to discharge	53.0 (90.8)	45.3 (74.9)	0.3	72.6 (92.2)	51.6 (118.5)	0.1
Time from Sx assess to first intervention	-	-	-	127.8 (501.6)	24.0 (28.1)	0.1

**Table 4:** Patient Outcome Variables for Acute Appendicitis and Acute Cholecystitis in the Pre-ACS (2007) versus Regionalized ACS (2011) Cohorts *Fisher’s Exact Test was used. ACS: Acute Care Surgery

Variable	Acute Appendicitis (AA)			Acute Cholecystitis (AC)		
	Pre-ACS n (%)	R-ACS n (%)	p-value	Pre-ACS n (%)	R-ACS n (%)	p-value
Risk of Perforated Appendicitis	40 (18.9)	94 (29.1)	0.006	-	-	-
Intra-Operative Complication Rate	1 (0.4)	3 (0.9)	0.9	12 (7.7)	7 (2.8)	0.02
Post-Operative Complication Rate by Grade						
Grades I & II	20 (8.2)	24 (7.3)	0.7	13 (8.3)	16 (6.4)	0.5
Grades III, IV, V	4 (1.6)	29 (7.5)	0.2	11 (7.0)	23 (9.1)	0.5
30-Day Readmission Rates	2 (0.8)	15 (4.6)	0.01*	6 (3.8)	19 (7.5)	0.1
30-Day Emergency Department Visits	8 (3.3)	14 (4.3)	0.6	10 (6.4)	15(5.9)	0.9

For patients with AA, regionalization of the ACS service did not result in significant improvements from the Pre-ACS service for transfer time, time from operating room to discharge, or total length of stay (LOS). Time to emergency room physician (ERP) for AA patients was significantly higher in the R-ACS as compared to the Pre-ACS cohort (3.4 ± 2.3 hours vs 2.4 ± 2.6 hours, p<0.001). However, these same patients experienced a significantly shorter time to surgical assessment/surgical response time (1.3 ± 1.2 hours vs 2.6 ± 4.3 hours, p<0.0001) as well as time from ERP or surgical assessment to imaging (4.1 ± 4.1 hours vs 6.9 ± 9.9 hours, p<0.0001). In addition, the frequency of pre-operative imaging (ultrasound (US), computed tomography (CT), magnetic resonance imaging (MRI)) was significantly higher among R-ACS AA patients as compared to Pre-ACS AA patients (93.3% vs 70.1%; p<0.0001). Similarly, the R-ACS service significantly reduced both time to surgical assessment/surgical response time (1.8 ± 1.5 hours vs 4.1 ± 5.0 hours, p<0.0001) and time from ERP or surgical assessment to imaging for those patients with AC (7.8 ± 1.9 vs 13.2 ± 18.5, p<0.0001). All other EOC outcome variables were unaffected under R-ACS services. Data collected from multiple time points pre- and post-ACS implementation were used to retrospectively assess for temporal trends in the data, similar to an interrupted time series analysis. Evaluation of length of stay (LOS) for the AA and AC patient groups confirmed the absence of pre-existing trends towards decreased LOS prior to the implementation of ACS and no additional temporal trends were identified. Table 4 presents a summary of the findings for the patient outcome variables assessed. Under both the Pre-ACS and R-ACS service models, no differences were found in the time to the operating room for patients with perforated and non-perforated AA. However, the R-ACS cohort demonstrated significantly higher rates of risk of perforated appendicitis (29.1% vs 18.9%, p<0.006) and 30-day readmission (4.6% vs 0.8%, p<0.01) when compared to the Pre-ACS service model (Table 4). Risk of perforated appendicitis was based on appendectomy pathology findings. The frequency of intraoperative complications for AA patients was the same for both the Pre- and R-ACS service cohorts. For AC patients, the R-ACS service significantly reduced the rate of intraoperative complications (2.8% vs 7.7%, p<0.02). The frequency of 30-day emergency department visits was similar between the Pre-ACS and ACS service models for both AA and AC patient groups.

Multivariate logistic regression demonstrated that post-operative complications and/or readmission for AA was associated with age, laparoscopic surgical procedure, transfer from another hospital, and admission to VGH or CGH as compared to SBGH (Table 5). Perforated appendicitis was associated with patient age and being transferred from another hospital versus direct admission (Table 6). Total LOS for AA patients was significantly associated with having a perforated appendix and open surgery versus laparoscopic surgery (Table 7). Time to operating room for AA patients was not significantly affected by age, gender, BMI, comorbidities, pre-operative imaging, and transfer to admitting hospital. For AC patients, the risk of post-operative complications and/or readmission was associated with age and having a laparoscopic procedure versus open surgery (Table 5). For AC patients, older age, presence of comorbidities, and longer time to operating room was associated with total hospital LOS and absence of a pre-operative intervention was the only covariate significantly associated with time to operating room (Table 8).

**Table 5:** Logistic Regression Model for Appendicitis and Acute Cholecystitis Post-Operative Complications and Readmissions ^1^OR=Odds Ratio; CI=Confidence Interval. ^2^VGH= Victoria General Hospital; CGH=Concordia General Hospital; SBGH=St. Boniface General Hospital.

	Acute Appendicitis		Acute Cholecystitis	
Variable	OR (95% CI)^1^	p-value	OR (95% CI)^1^	p-value
Age	1.0 (1.0, 1.0)	0.01	1.0 (1.0, 1.1)	0.001
Gender				
Male	1.6 (0.9, 2.9)	0.1	0.8 (0.4, 1. 5)	0.4
Female	reference	-	-	-
Laparoscopic versus Open				
Laparoscopic	0.4 (0.2, 0.8)	0.01	0.2 (0.1, 0.5)	0.001
Open	reference	-	reference	-
Transfer versus Direct Admit				
Transfer	2.0 (1.0, 3.9)	0.04	-	-
Direct	reference	-	-	-
Admission Hospital^2^				
VGH/CGH	2.4 (1.1, 5.1)	0.03	0.5 (0.2, 1.2)	0.1
SBGH	reference	-	reference	-
Operating Room Time of Day				
Day	-	-	reference	-
Evening	-	-	1.7 (0.9, 3.1)	0.1
Night	-	-	1.8 (0.5, 6.3)	0.4

**Table 6:** Logistic Regression Model for Perforated Appendix ^1^OR=Odds Ratio; CI=Confidence Interval.

Variable	OR (95% CI)^1^	p-value
Age	1.0 (1.0, 1.0)	<0.0001
Gender		
Male	0.9 (0.6, 1.4)	0.7
Female	reference	-
Transfer versus Direct Admit		
Transfer	1.7 (1.1, 2.6)	0.01
Direct	reference	-
Operating Room Time of Day		
Day	reference	-
Evening	0.7 (0.4, 1.15)	0.2
Night	1.22 (0.7, 2.2)	0.5

**Table 7:** Linear Regression Model for Hospital Length of Stay for Appendicitis ^1^OR=operating room; BMI: body mass index. ^2^N=416 observations; r^2^=0.12 (statistically significant).

Parameter^1, 2^	Estimate	Standard Error	t Value	Pr > |t|
Intercept	5968.6	1487.0	4.0	<0.0001
Age	15.3	13.0	1.2	0.2
Gender - Female	-491.9	393.0	-1.3	0.2
Gender - Male	0.0	.	.	.
BMI	0.8	35.0	0.02	1.0
Comorbidity - 0	-1184.1	659.5	-1.8	0.07
Comorbidity - 1	0.00	.	.	.
Perforated – 0	-2419.2	462.0	-5.2	<0.0001
Perforated - 1	0.00	.	.	.
Type of OR procedure– Open	2064.9	712.0	2.9	0.004
Type of OR procedure– Laparoscopic	0.00	.	.	.
Time to OR	0.6	0.5	1.1	0.3
Pre-operative intervention	0.00	.	.	.
OR time of day – Day	-151.6	598.4	-0.3	0.8
OR time of day – Evening	-462.8	541.1	-0.9	0.4
OR time of day – Night	0.00	.	.	.
Admission hospital – St. Boniface	-468.9	616.1	-0.8	0.5
Admission hospital – VGH/CGH	0.0	.	.	.

**Table 8:** Linear Regression Model for Hospital Length of Stay for Acute Cholecystitis ^1^OR=operating room; BMI: body mass index

Parameter	Estimate	Standard Error	t Value	Pr > |t|
Intercept	-4425.2	3798.0	-1.2	0.3
Age	114.6	25.7	4.5	<0.0001
Gender – Female	1205.3	811.8	1.5	0.1
Gender – Male	0.0	.	.	.
BMI	62.3	58.5	1.1	0.3
Comorbidity – 0	-2936.1	1031.7	-2.9	0.005
Comorbidity – 1	0.0	.	.	.
Type of OR^1^ procedure – Open	3885.9	2124.9	1.8	0.07
Type of OR procedure– Laparoscopic	0.0	.	.	.
Time to OR	1.7	0.2	7.0	<0.0001
Pre-operative intervention - 0	864.5	1129.5	0.8	0.5
Pre-operative intervention - 1	0.0	.	.	.
OR time of day – Day	-301.3	1790.7	-0.2	0.9
OR time of day – Evening	-573.1	1771.8	-0.3	0.8
OR time of day – Night	0.0	.	.	.
Admission hospital – St. Boniface	310.8	1244.2	0.3	0.8
Admission hospital – VGH/CGH	0.0	.	.	.

## Discussion

This study assessed multiple EOC and patient-related outcomes for acute appendicitis and acute cholecystitis following the implementation of an R-ACS service in Winnipeg (Manitoba, Canada).

The R-ACS in Winnipeg resulted in not only some improvements in efficiency but also highlighted some areas for improvement. Time to ER physician assessment increased but time to surgical assessment/surgical response time and time to imaging decreased, indicating improved efficiency by a dedicated surgical team. With R-ACS, time to surgery was slightly increased for AA and did not improve for AC, highlighting the challenge of timely access to operating room resources for EGS patients even with the presence of a dedicated team. Time to discharge and LOS trended towards improvement. Postoperative care was already efficient under the traditional model, with the average LOS between 1.4 to 2.8 days (depending on simple versus complex appendectomy) for AA and 2.9 days for AC, and improvements in this area were not demonstrated. Mean LOS for AA and AC from 2011 to 2017 were one and three days, respectively; one day less than the two additional Winnipeg hospitals performing these procedures. Multiple factors have been shown to impact LOS for AA patients, including time of presentation, perforation, and time of surgery [21-22].

Currently, there are no established EOC benchmarks for common EGS diagnoses. The development of wait-time benchmarks for common EOC measures is required to promote patient safety and inform program planning and evaluation.

Winnipeg has historically reported some of the longest ER wait times in Canada. The Canadian Institute for Health Information (CIHI) reported that four Winnipeg emergency departments were among the top five, with the longest wait times in Canada [23]. Multiple competing factors influence ERP assessment time, including ER patient flow, delays in admissions/ER clearance, and increased patient volumes. It is difficult to weigh the influence of various factors on the overall increase in ERP assessment time shown in this study without assessing the impact of each individual factor in detail. A comprehensive assessment of ER flow was beyond the scope of this study. However, significant restructuring of emergency care is underway in Winnipeg as part of an overall regional reorganization strategy. The impact of these changes will need to be assessed in the future.

We assessed intraoperative and perioperative patient outcomes related to AA and AC using commonly reported measures and classifications. Despite trainee involvement in the R-ACS services model, post-operative complications did not increase and there were significantly fewer intra-operative complications for AC and no change for AA. The same surgeons were involved in the pre-ACS and R-ACS cohorts but the reduction in complications for AC might be explained by increased experience with laparoscopy during the study period or possibly by the conduct of these procedures at a tertiary teaching institution. Variation in study definitions and outcome measures for EGS conditions are common throughout the scientific literature [17]. This makes a direct comparison among study results challenging. The standardization of common outcome measures both intraoperatively and perioperatively is necessary.

In this study, R-ACS resulted in a substantial increase in patient transfers, with 67.1% of appendectomies being transferred, as compared to only 14.3% in the Pre-ACS cohort. These patients were at higher risk of pathologically confirmed perforation and post-operative complications (or readmission). At 29%, the R-ACS AA perforation rate is at the high end of published national rates of 20%-30% [24]. Other ACS studies have reported rates of 4.6% [14] and 17% [15] and a reduction of 10% [25]. Other studies have reported increased perforation rates with ACS service model implementation [15-16].

The risk of perforation might be influenced by delays in imaging or consultation requests at the origin hospital, inadequate initial care (antibiotics and fluid resuscitation), or as the result of more serious disease. We did not capture index hospital treatment and efficiencies as part of the current study. This would involve significant resources to review paper-based emergency department data at multiple sites, including transfers from outside of Winnipeg. This has been considered for future research.

Short delays in surgical intervention for AA are well-tolerated, with several studies reporting no increase in rupture when surgery is delayed until daytime hours [26-27]. One study reported the risk of rupture as negligible within the first 36 hours after symptom onset, with the risk rising 5% for each 12-hour period with untreated symptoms [28]. R-ACS assessment of patients was faster but the number of daytime operations did not increase. This is likely as a result of competing surgical procedures, such as complex emergency bowel cases, which were more likely to be scheduled in daytime operating hours. In addition, fewer AA patients had evening surgery and more had night-time surgery with R-ACS. It is likely that appendectomies were performed at night out of necessity due to an increased volume of cases rather than the influence of surgeon remuneration. Within the ACS literature, most studies report increased daytime operating with ACS services while only one study found no increase in daytime operating [12-14]. Our logistic regression analysis did not show the operative time of day to be associated with patient outcomes. The increased 30-day readmission rate for AA in the R-ACS cohort is likely associated with the increased rate of appendicular rupture. While some of these patients were treated with antibiotics, a significant number required either percutaneous or operative drainage.

Our study has a few limitations. Firstly, while transfer time was similar among the Pre-ACS and R-ACS cohorts, we did not capture the time spent at the referring hospital or treatments administered at the initial hospital. Secondly, our pre/post-study design lacked a concurrent control/comparison group, making it difficult to establish a cause and effect relationship between the intervention (ACS service implementation) and the outcomes measured. Thirdly, our results may be confounded by temporal and secular changes independent of the intervention. Surgeons who participated in EGS calls under the traditional (Pre-ACS) service model of care were the same as those involved in the R-ACS model and their laparoscopic experience increased over time.

Regression analysis showed that laparoscopic cases were associated with a decreased risk of postoperative complications, decreased readmissions, and shorter LOS. In the last five years, early surgical intervention for AC has been advocated and the hospitals providing the R-ACS service adopted this change. However, our results show no significant differences in time to OR or operative rate for AC between the Pre-ACS and R-ACS cohorts. Other temporal trends in practice patterns have not been captured by this study (favored antibiotic regimens, changes in antibiotic resistance). Also, competing procedures for the emergency OR were not assessed and could have impacted the R-ACS daytime OR measure.

The introduction of formal ACS services, along with changes in surgeon practice patterns across Canada, make the further regionalization of EGS likely, and its impact must be highlighted. As with other conditions with standardized protocols for care (myocardial infarctions or traumas) [28], pre-transfer guidelines, and standardized inter-hospital documentation could potentially improve the timeliness and quality of care. Some Canadian centers have developed clinical care pathways for patients with common EGS diagnoses [29]. A “Suspected Appendicitis Care Map” for adults has been developed to streamline the flow of patients from the ED to the OR [28].

## Conclusions

EGS patients are a uniquely challenging population, particularly prone to poor outcomes. Improved efficiency and quality of EGS care can significantly impact the emergency department, operating room, and hospital resources. We must continue to define, measure, and monitor specific outcomes in order to identify opportunities for improvement.

## Appendices

**Table 9:** Supplemental File 1: Data Quality Form - Demographic Variables

Supplemental File 1		
Data Quality Form – Demographic Variables		
Data Collected	Location in Chart	Quality
Diagnosis	Face sheet	Electronic
Origin hospital	Face sheet	Electronic
RHA of origin hospital	N/A	N/A
Admission hospital/service	Face sheet	Electronic
Age at admission	Face sheet	Electronic
Gender	Face sheet	Electronic
BMI	2007: Anesthesia pre-op checklist 2011: EPR	2007: Hand-written 2011: Electronic
Comorbidities	ERP assessment Surgery consult Anesthesia consult	Handwritten
Previous laparotomy	ERP assessment Surgery consult Anesthesia consult	Handwritten
Smoking status	ERP assessment Surgery consult Anesthesia consult Anesthesia pre-op checklist	Handwritten
Medications	DPIN	Electronic
Previous admission for same diagnosis (date, location)	ERP assessment Surgery consult Previous admissions listed in chart	Handwritten 2011: EPR

**Table 10:** Supplemental File 2: Data Quality Form - Intervention Variables

Supplemental File 2		
Data Quality Form – Intervention Variables		
Data Collected	Location in Chart	Quality
Non-Operative Interventions		
Type	Procedure note (eg, ERCP)	Electronic
Date Performed And Time Performed	Nursing procedure record	Hand-written
Location Performed	Procedure note	Electronic
Ordered By	Orders Consult Requisition	2007: Handwritten 2011: EPR
Radiology		
Type and Date Performed	Radiology report	Electronic
Time Performed (Start time)	2007: Nursing notes, radiology technician reports (progress notes) 2011: Radiology report	2007: Handwritten 2011: EPR
Location Performed	Radiology report	Electronic
Ordered By	Orders Requisition Radiology report	2007: Handwritten 2011: EPR
OR		
Type	OR dictated report	Electronic
Date, Start Time, End Time	Nursing OR record	Handwritten
Length of OR and Time of Day	Calculated	N/A
Drain/Conversion	OR dictated report	Electronic
Pathology	Pathology report	Electronic
Excel Calculation: (((End_Date+End_Time)-(Start_Date+Start_Time))*24)*60		

**Table 11:** Supplemental File 3: Data Quality Form - Efficiency of Care

Supplemental File 3		
Data Quality Form - Efficiency of Care		
Data Collected	Location in Chart	Quality
Referring Facility	Face Sheet Photocopied notes from referring facility	Electronic
Consult Date	Consult Nursing notes ACSS Transfer Form*	Handwritten Handwritten Handwritten
Consult Time	Consult Nursing notes ERP assessment ACSS Transfer Form* Fax time of transfer information	Handwritten Handwritten Handwritten Handwritten Electronic
Assessment Room Date/Time	Nursing notes	Handwritten
Triage Time	Triage sheet	Electronic
Admit Date, Admit Time, Discharge Date	Face Sheet	Electronic
Discharge Time	Face Sheet Nursing notes	Electronic Handwritten
ERP Assess Date	ERP Assessment	Electronic
ERP Assess Time	ERP Assessment Nursing Notes Orders	Handwritten Handwritten EPR (2011)
ERP-Sx Consult Date	Consult	Handwritten, EPR* (2011)
ERP-Sx Consult Time	ERP Assessment Consult Nursing Notes	Handwritten Handwritten, EPR* (2011) Handwritten
Sx Assess Date	Consult	Handwritten
Sx Assess Time	Consult Orders Nursing Notes	Handwritten Handwritten, EPR (2011) Handwritten
*Inconsistently present; Excel Calculation: (((End_Date+End_Time)-(Start_Date+Start_Time))*24)*60		
